# Active transport between home and school assessed with GPS: a cross-sectional study among Dutch elementary school children

**DOI:** 10.1186/1471-2458-14-227

**Published:** 2014-03-05

**Authors:** Dirk Dessing, Sanne I de Vries, Jamie MA Graham, Frank H Pierik

**Affiliations:** 1 TNO, Department of Life Style, Leiden, The Netherlands; 2 TNO, Department of Urban Environment and Safety, Utrecht, The Netherlands; 3 Department of Public & Occupational Health and EMGO+ Institute, VU University Medical Center, Amsterdam, The Netherlands; 4 Research group Healthy Lifestyle in a Supporting Environment, The Hague University of Applied Sciences, Johanna Westerdijkplein 75, The Hague, The Netherlands

**Keywords:** Elementary school, Children, Global positioning system (GPS), Mode of transport, Walking, Cycling

## Abstract

**Background:**

Active transport to school is associated with higher levels of physical activity in children. Promotion of active transport has therefore gained attention as a potential target to increase children’s physical activity levels. Recent studies have recognized that the distance between home and school is an important predictor for active travel among children. These studies did not yet use the promising global positioning system (GPS) methods to objectively assess active transport. This study aims to explore active transport to school in relation to the distance between home and school among a sample of Dutch elementary school children, using GPS.

**Methods:**

Seventy-nine children, aged 6-11 years, were recruited in six schools that were located in five cities in the Netherlands. All children were asked to wear a GPS receiver for one week. All measurements were conducted between December 2008 and April 2009. Based on GPS recordings, the distance of the trips between home and school were calculated. In addition, the mode of transport (i.e., walking, cycling, motorized transport) was determined using the average and maximum speed of the GPS tracks. Then, proportion of walking and cycling trips to school was determined in relation to the distance between home and school.

**Results:**

Out of all school trips that were recorded (n = 812), 79.2% were classified as active transport. On average, active commuting trips were of a distance of 422 meters with an average speed of 5.2 km/hour. The proportion of walking trips declined significantly at increased school trip distance, whereas the proportion of cycling trips (β = 1.23, p < 0.01) and motorized transport (β = 3.61, p < 0.01) increased. Almost all GPS tracks less than 300 meters were actively commuted, while of the tracks above 900 meters, more than half was passively commuted.

**Conclusions:**

In the current research setting, active transport between home and school was the most frequently used mode of travel. Increasing distance seems to be associated with higher levels of passive transport. These results are relevant for those involved in decisions on where to site schools and residences, as it may affect healthy behavior among children.

## Background

Being sufficiently physically active is important for children. It is associated with a wide range of health benefits. For example, being physically active is related to improved cardiovascular risk factors, enhanced bone mineral density and improved psychological well-being [1,2]. At present, when children’s physical activity is compared to recommendations made by the WHO [3] or Dutch Standards for Healthy Activity [4,5], the low number of children that are sufficiently physically active is alarming. The WHO recommends children to be physically active for at least 60 minutes each day. International [6] and Dutch [7] research concluded that only 30-40% of the children currently meet this recommendation. Consequently, stimulating children to be physically active is part of current health promotion and disease prevention strategies. In this regard, the journey between home and school is gaining more and more attention as a potential source of physical activity for children [8-11]. For example, a study by Cooper et al. [12] showed that for children that used active transport, the journey to school could contribute towards reaching daily physical activity requirements. Besides, not only children benefit from this, it could also be beneficial to people who live in proximity to the school. Traffic congestion in the areas surrounding schools can be significant. Furthermore, the reduction of motorized transport to school is likely to have a positive influence on the local environment, for example by reducing regional air pollution levels [13,14]. Unfortunately it appears that, in the past decades, the number of children that actively commute to school has steadily declined. For example, the proportion of students in the US that walk or bike to school has dropped from around 40% in 1969 to 12% in 2007 [15]. Similar numbers have been observed in Australia [16]. Although the proportion of children that actively commute is generally much higher in Europe, there is some evidence that suggests the proportion of active travel is also declining in European countries [17]. Moreover, it is expected that changes in mode of transport will be different in countries where cycling is much more common (e.g. Denmark, Germany, the Netherlands) [18].

In recent research a wide spectrum of correlates for active commuting has been identified, ranging from individual and family factors, school characteristics, social factors to physical environmental factors [19,20]. Of these correlates the reported distance to school seems to be the strongest predictor of using active rather than passive transport [21]. Literature shows that when the reported distance increases, the probability of active travel to school decreases [22-26]. The measurement of the distance traveled differs between studies. Most studies have used self-reported distance, others have calculated the shortest possible route along the road network by using geographic information systems (GIS). GIS estimations of the shortest route have the advantage that they do not suffer from recall bias by the respondents. On the other hand, they still do not always accurately reflect the actual traveled route [27]. A more accurate way to monitor the route to school is by the use of GPS. Moreover, there is also no standardized method for measurement of mode of travel [21]. Some studies used parents’ estimates of their children’s frequency of walking and cycling to school, while others relied on self-reports from children, or used independent observers to report the mode of transport. Finally, although both walking and cycling are considered active forms of transport, they are two distinct modes of transport that should be separated in the analysis since they have been associated with different correlates in previous research [28,29].

The use of global positioning systems (GPS) might be the solution to the previously mentioned issues associated with measurement of distance and assessment of mode of transport. Recent technological advancement in GPS receivers has made them easy to carry, and these devices have already been used for measuring free-living activities in children without interfering with their day-to-day activities [30,31]. Thus, the use of GPS offers great potential for progress in the field of health research [32] and brings a new and objective method to monitor the actual distance traveled *and* mode of transport of children.

The aim of the present study was to analyze the relationship between the distance between home and school and the proportion of children actively traveling to school, using data collected with GPS receivers. With objective data, better informed decisions on potential school siting can be made, or other policy measures that might encourage active transport among school children.

## Methods

### Participants and setting

This study was part of the SPACE (Spatial Planning And Children’s Exercise) study, in which the relationship between physical environmental characteristics and children’s physical activity was investigated [26,33].

Out of the twenty elementary schools that participated in the SPACE study, a convenience sample of six schools was selected to take part in this study. These six schools were located in five different Dutch cities with >70.000 residents (i.e. Amersfoort, Rotterdam, Hengelo, Haarlem and Vlaardingen). The schools are situated in neighborhoods that are demographically similar (i.e. age distribution, social economic status, ethnicity) and contain comparable types of buildings (i.e. residence type, year of construction).

Elementary school children, between the ages of 5 and 11 years old, were invited to take part in the study through letters and pamphlets that were handed out by teachers of the participating schools. This resulted in a group of 97 children that were all asked to wear GPS receivers for one week. All children lived in the same neighborhood as the school they attended. Furthermore, informed consent was obtained from a parent or guardian for all children that took part in the study. The measurements were conducted between December 2008 and April 2009, and temperatures during the recording period ranged between 1 and 6 degrees Celsius. The study was approved by the ethics committee (IRB) of the Leiden University Medical Center.

### Instrumentation / measures

All children were requested to wear a GPS receiver (Travel recorder X, BT-Q1000X, QStarz International Co) on awakening in the morning until bedtime for eight consecutive days during a regular school week. The GPS receivers were set to record the geographical position of the children with a sampling frequency of 5 seconds. Children were instructed on the use of the GPS receivers when these were handed out during school hours. The GPS was attached to the children’s waist with an elastic belt. During activities where the GPS receiver could be damaged, or uncomfortable to wear, the children were asked to temporarily remove the device (e.g. during swimming, showering, sleeping). To ensure parents and children could read back all instructions, a manual was handed out with the device.

In addition, the children’s body height (without shoes and socks) and body weight (with clothes, without shoes) were measured with a microtoise (Stanley 04-116) and a digital scale (Seca 812, Vogel & Halke GmbH & Co) respectively. These measures were used to calculate BMI (kg/m^2^), and to categorize the children into ‘normal weight’ or ‘overweight’ according to age- and sex-specific cut-off points for children by Cole et al. [34]. All children that were above normal weight, including the obese, were classified as ‘overweight’. Furthermore, a parent or guardian completed a questionnaire to provide information on children’s age, gender, home address, and other demographic variables.

### Data handling

**Figure 1:**
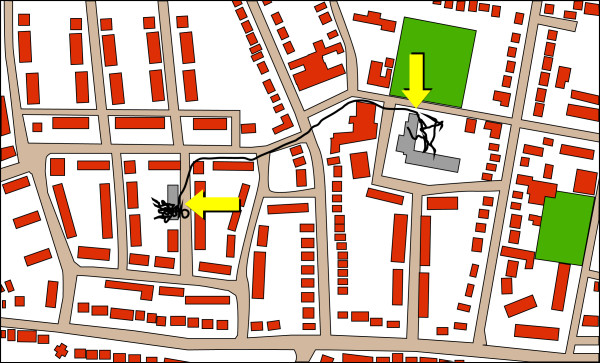
**Example of a child traveling from home towards the school building.** Description: The left arrow indicates the start of the track at the home address, the right arrow indicates the end of the track at the school building.

All GPS data were downloaded to a computer with Q-Travel, a travel data management software package from Qstarz, and then mapped with the URBIS III software package [35]. First, the location of the home address and the school building of the children were determined based on the GPS-data. This was done based on a cluster detection method which can be used to distinguish indoor and outdoor activity [36]. Cluster detection was used because geocoding of the home and school addresses based on postal codes can result in inaccuracy [37]. The straight line distance between the children’s home address and the school location was then calculated using the Pythagorean theorem. Second, for all children, each GPS track between the home address and the school location was identified with an automatic procedure in URBIS. The procedure to recognize these tracks started by identifying the first recorded GPS point that was located within 25 meters of the home address. Next, the recorded GPS data was searched chronologically until the first recorded GPS point was detected that was located within 25 meters of the school building. If a GPS point within 25 meters of the home location was detected first, this was used as the new starting point of the track. All data points between the two GPS points were considered to be part of the GPS track between home and school. Once a track between the two points of interest was identified, it was extended in both directions as close as possible to the actual location of the address. An example of such a track is shown in Figure 1. Once all relevant tracks starting from the home location were identified, the whole procedure was repeated, but this time starting with all GPS points within 25 meters of the school building. This way, tracks going from school towards the home location could also be identified. Tracks going in either direction (i.e. home or school) were included in the analysis. For each of these tracks *total distance*, *time duration*, *average speed* and *maximum speed* were computed. To reduce the chance that the automated procedure incorrectly selected tracks that were not situated between the home and school location, all GPS tracks whose distance or duration deviated from the standard deviation within and between subjects were detected. This was done separately for active and passive transport tracks. All tracks that deviated beyond three times the standard deviation ± the mean were removed from the analysis.

Third, the mode of transport was determined for each track using the average and maximum speed. In the present study, the following cut-off points were used: when the average speed of the track was below 10 km/h and the maximum speed was below 14 km/h, the track was categorized as ‘walking’. When the average speed of the track was below 25 km/h and the maximum speed was below 35 km/h, the track was categorized as ‘cycling’. All remaining tracks with maximum speeds under < 150 km/hour were categorized as ‘motorized transport’. These values are similar to the cut-off points used by Bohte and Maat [38]. Next, descriptive statistics of the tracks (average distance, average time duration, average speed and maximum speed) were calculated separately for each mode of transport. Furthermore, the distribution of the mode of transport was displayed in relation to the distance of the tracks. This was done by calculating the percentage of walk, cycle or motorized transport tracks for distance categories of 100 meters.

### Statistical analysis

A multinomial logistic mixed-effects model was used to assess whether the distance of the GPS track was related to the mode of transport of the children. This model was used to account for the clustered data structure where clusters of children lived in the same city and where individual children recorded a different number of tracks. Distance of the recorded track was used as the independent variable, mode of transport was the dependent variable. The analysis was done on the level of the GPS tracks. This way, the hypothesis was tested whether GPS tracks of relatively larger distance had a higher chance to be classified as passive transport. Within this model, this meant that the tracks defined as ‘walking’ were used as a reference category to estimate the relative odds of a track being either ‘cycling’ or ‘motorized transport’ as distance of the track increased. Since distance was not normally distributed, a logarithmic transformation was performed before it was used in the model. To examine whether the mode of transport was comparable for going home and going to school, the direction of the track (i.e. towards home or school) was also added to the model, using ‘going towards home’ as the reference category. Results were adjusted for city, gender and age of the children. All analyses were performed in SPSS version 20.0.

## Results

**Figure 2:**
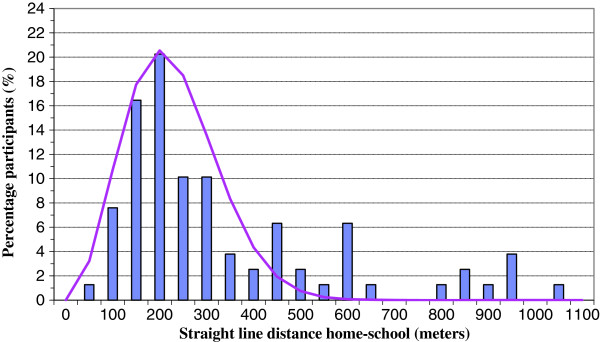
Distribution of the straight line distances between home and school.

Of the 97 children that carried a GPS receiver, 86 children recorded one or more tracks between home and the school building. Children (n = 3) that lived very close to the school building (< 50 meters) were excluded because the inaccuracy of GPS recordings (10-15 meters) has a relatively large influence on such short distances. After removal of tracks that deviated in distance, duration or maximum speed, the final study population consisted of 79 participants that recorded 812 GPS tracks for further analysis. The age of the children ranged between 5 and 11 years, with an average of 8.6 (SD ± 1.40) years. Average BMI of the final study population was 18.2 (SD ± 3.3) kg/m^2^. Further descriptive statistics of the population are shown in Table 1.Figure 2 shows the distribution of the (straight line) distance between the home address and the school building among the study population. The distribution is slightly skewed to the right: most of the children live within a perimeter of 500 meters from the school building. Average distance between home and school is 364 meters, median distance is 268 meters.

**Table 1:** Descriptive statistics of the population

	**Total**		**Boys**		**Girls**	
	***n***	***Mean *** **± *SD***	***n***	***Mean *** **± *SD***	***n***	***Mean *** **± *SD***
*Age (years)*	79	8.6 ± 1.4	37	8.6 ± 1.3	42	8.5 ± 1.4
*Body height (cm)*	78	137.5 ± 9.6	36	138.0 ± 9.3	42	137.0 ± 10.0
*Body weight (kg)*	78	34.8 ± 10.3	36	34.8 ± 9.5	42	34.9 ± 10.9
	***n***	***%***	***n***	***%***	***n***	***%***
BMI category						
*Normal*	53	67.9	26	72.2	27	64.3
*Overweight*	25	32.1	10	17.8	15	35.7
City						
*Haarlem*	8	10.1	2	5.4	6	14.3
*Amersfoort*	30	38.0	16	43.2	14	33.3
*Hengelo*	24	30.4	12	32.4	12	28.6
*Vlaardingen*	8	10.1	4	10.8	4	9.5
*Rotterdam*	9	11.4	3	8.1	6	14.3

On average, participants recorded 10.3 tracks (SD ± 6.6) between the home address and the school building (and vice versa). Out of the 812 tracks, 44.6% were classified as walking, 34.6% as cycling and 20.8% as motorized transport. Thus, 79.2% of the recorded tracks were classified as active transport. Of all children, 92% (n = 73) recorded at least one trip to school that was classified as either walking or cycling. When children walked or cycled, they recorded an average track distance of 474 meters between home and school. On average, their journey between home and school took 8 minutes at an average speed of 5.2 km/h. Further descriptive statistics for the travel modes (i.e. distance, time duration, average speed and maximum speed) are shown in Table 2. Out of the 812 tracks in the analysis, more than half of the tracks (n = 434) were tracks from school towards the home address. There was no significant difference in mode of transport between the two directions, i.e. going home or going towards school (β = 0.37, p = 0.24). Most of active transport took place within a range of 200 and 600 meters. At distances of beyond 1500 meters, very few active transport tracks were recorded.

**Table 2:** Average distance, time duration and speed of GPS tracks between home and school

**Mode of transport**		**Mean ± SD**	**Median**
*Walking*	Distance (meter)	357 ± 264	288
*N = 362*	Duration (minutes)	6.9 ± 8.9	4.2
	Average speed (km/h)	4.1 ± 1.3	4.1
	Max speed (km/h)	9.9 ± 2.4	10.1

*Cycling*	Distance (meter)	624 ± 383	560
*N = 281*	Duration (minutes)	8.4 ± 9.1	4.9
	Average speed (km/h)	6.8 ± 3.8	5.6
	Max speed (km/h)	19.2 ± 4.8	17.4

*Motorized transport*	Distance (meter)	2941 ± 4208	1176
*N = 169*	Duration (minutes)	42.3 ± 72.8	7.4
	Average speed (km/h)	10.6 ± 7.8	9.1
	Max speed (km/h)	53.6 ± 18.6	48.8

Description: N represents the number of tracks recorded for the different modes of transport.

**Figure 3:**
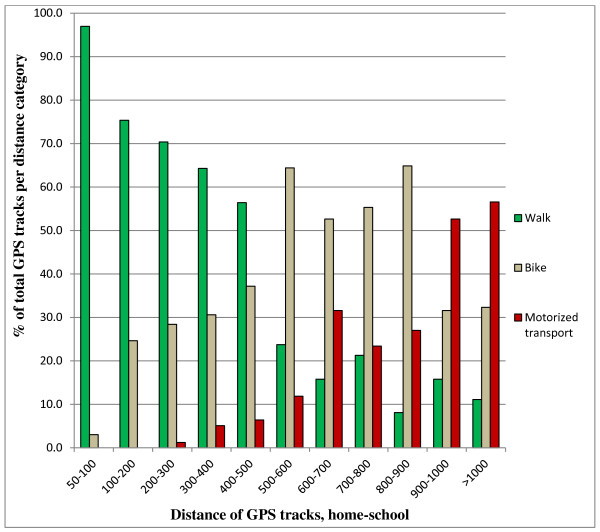
Mode of transport distribution in relation to distance of the track (in categories of 100 meters).

Figure 3 shows how the mode of transport is distributed among the different track lengths. Since most children lived within a 500 meter perimeter around their school, the number of recorded tracks is distributed unevenly over the distance categories. For example, only 22 tracks were recorded between 900 and 1000 meters, while there are 162 tracks in the category 200-300 meters. As can be seen in the figure, almost no tracks (n = 2) were classified as motorized transport within a distance below 300 meters. In contrast, of the tracks longer than 900 meters, more than half of the tracks were classified as motorized transport. There is a significant decrease in the percentage of walking tracks as the track distance increases. With ‘walking’ as the reference category, distance was significantly associated with an increase in cycling *(adjusted β = 1.23, p < 0.01, crude β = 1.24, p < 0.01)* and motorized transport *(adjusted β = 3.61, p < 0.01, crude β = 3.60, p < 0.01)*.

## Discussion

The aim of this study was to explore the relationship of the distance between home and school and the proportion of active transport trips. This study used objective methods to measure the distance and classify the mode of transport of children’s journey between home and school. Other studies that have measured the route during active transport relied on subjective recall of the trip length, e.g. [39], or on the use of GIS to calculate the distance of a route, e.g. [22]. These methods are considered to be less accurate. The actual route distance to/from school has not yet been assessed objectively in previous research that focused on the mode of transport [21].

In the current study almost all of GPS tracks with a distance below 300 meters were classified as active transport (99.2%), and out of the tracks with distances above 900 meters, more than 50% was classified as motorized transport. A significant decline in the proportion of children that walked could be observed with increased distance. Results of this study confirm the findings of previous studies [22,23,28,39] which also concluded that as the distance to school increases, the likelihood of active school travel decreases. For example, a study among Australian schoolchildren revealed that children were at least 5 times more likely to actively commute to school if their distance to school was shorter than 800 meters [22]. Most of the previous studies on active transport have been conducted outside of Europe where children generally live further from their elementary school and the distance between home and school tends to be longer. In the study from Yeung and colleagues [39] in Australia, active commuters traveled an average distance of 1.5 kilometers per trip whereas in the present study, almost all children lived within 1 kilometer of the school building. Average straight line distance between home and school was 364 meter, and active commuting trips had an average length of 422 meters. Our study showed that even when children live within a relative short distance from their school building (< 1 km), a decline in the proportion of children actively commuting to school can be observed with increasing distance of the school journey.

In the current study population, most of the tracks (79.2%) were classified as active transport. It is likely that this is an underestimation of the percentage of active transport trips in other seasons, since most of the measurements were conducted during the winter period [40]. The finding that the majority of children used active transport in the current study is in contrast with other studies that have found motorized transport to be the most frequently used mode of transport (e.g.[23]). This might be partly due to the fact that cycling is a very popular and practical method of transport in the Netherlands, especially when compared to the UK and US where only about 1% of trips are conducted by bicycle [18]. Results from the present study thus might not be applicable to populations of schoolchildren outside the Netherlands. Key to the high levels of cycling in the Netherlands seem to be the separate bicycle lanes, combined with traffic calming measures in residential neighborhoods [18]. Moreover, schools in the Netherlands are relatively close to their residents. Even in the least densely populated municipality, the average distance to an elementary school is smaller than 2 kilometer [41].

In this study, no difference was found in the mode of transport to school and the mode of transport on the way back home. Research by Mitra et al. [42] did reveal a moderating effect for time of day on the mode of transport to school. Parents are thought to find it convenient to drive to school and drop their children on their way to work, but cannot drive their children back home. Also, parents may worry about traffic danger and ‘stranger danger’ on the road to school. The darkness in the morning influences both road safety and social safety, making cycling or walking to school more dangerous [43]. In the Netherlands, with a high prevalence of vulnerable road users such as cyclists, road safety is also influenced by the phenomenon of ‘safety in numbers’ [44]. This means that because of the high percentage of active transport among school children, motorists adjust their behavior and thereby increase road safety. How these aforementioned barriers and facilitators are associated with active transport among children living in close proximity (<1 km) from their school needs further investigation.

### Strengths and limitations

A strength of this study is that we have automatically detected trips between the two points of interest (i.e. school and home). When collecting GPS data among children, distinguishing all tracks between home and school can be a time consuming process, even in the current study which had a relatively small sample size. Southward et al. [45] have done this by manually assessing all GPS tracks between 8 am and 9 am and tracks between 15 pm and 17 pm. With the automated method, all trips during the day, including trips during lunch breaks, could be used in the analysis. This method could be helpful in future research, for example when information from GPS-enabled mobile phones is unaccompanied by additional information (e.g. travel diaries) from students to code journeys as active or not. On the other hand, the use of the automatic process was the reason that some of the tracks between home and school included stop-overs at other destinations, for example a stop at a friend’s house or at extracurricular childcare facilities. This explains why distance and duration of some of the passive transport tracks is longer than expected solely based on the straight line distances between home and school. Next to trip detection, the mode of transport was also automatically classified in our study. This was done using the average and maximum speed of the recorded tracks. With this method, Bohte and Maat [38] could correctly classify the right mode of transport in around 70% of journeys. Results of the current study are thus expected to be influenced by similar inaccurate classification of transport mode. Unfortunately, the additional validation system that they suggest could not be applied to the present study because data collection took place in 2009. In this validation system parents are requested to give additional feedback after the classification of transport mode is made. In future studies the classification accuracy might be further improved by using such automated validation systems and integrating GPS data with other objectively recorded data, for example with data from accelerometers. This combined method may also be used to distinguish active cycling from passive transport on the bicycle (i.e. children sitting on the back of the parent’s bicycle). Recently, a Danish research group has successfully classified transport to school into active or passive with the use of accelerometers [46]. The combination of accelerometers and GPS might offer a reliable way to further refine the classification of transport mode, for example by identifying types of activity through the use of neural networks [47], but these methods need to be validated and refined in future research.

## Conclusions

In conclusion, within the current research setting with a relatively small sample of elementary school children, there was a significant relationship between the distance between home and school and the transport mode. Active transport was the most frequently used mode of travel, and with increasing distance between home and school, significantly higher proportions of motorized transport were observed. Future studies should investigate whether the results found in our study can be generalized to older children, children living in other countries, more rural areas or areas with different urban form. Meanwhile, urban planners should realize that distance to school seems to be important when designing the mobility infrastructure and when planning housing and potential school sites. Unfortunately, this is not as easy as it might seem. Distance between home and school is shaped by complex social and economic processes that influence the location of the home and school address [21]. Understanding of these processes might provide ways to stimulate active transport among school children [28]. Furthermore, it should be realized that, although distance between home and school seems to be an important correlate associated with children’s transport mode, there are other correlates that are also associated with transport mode, such as individual, family, school, social and physical environmental factors.

## Competing interests

The authors declare that they have no competing interests.

## Authors’ contributions

DD carried out data analysis, interpretation of data and wrote the manuscript. SIV provided overall supervision and review for the present manuscript, was responsible for study conception, and was involved with data acquisition and study design. JG conducted preliminary data analysis, was involved in interpretation of data and approved the manuscript. FP helped conceive the study, was involved with data acquisition and revised the manuscript critically. All authors read and approved the final manuscript.

## Pre-publication history

The pre-publication history for this paper can be accessed here:

http://www.biomedcentral.com/1471-2458/14/227/prepub
